# A new FGFR inhibitor disrupts the TGF‐β1‐induced fibrotic process

**DOI:** 10.1111/jcmm.14793

**Published:** 2019-11-06

**Authors:** Mi‐Hyoung Kim, Seung‐Youn Jung, Kyung‐Hee Song, Jeong‐In Park, Jiyeon Ahn, Eun‐Ho Kim, Jong Kuk Park, Sang‐Gu Hwang, Hee‐Jong Woo, Jie‐Young Song

**Affiliations:** ^1^ Division of Radiation Biomedical Research Korea Institute of Radiological & Medical Sciences Seoul Korea; ^2^ Laboratory of Immunology College of Veterinary Medicine Seoul National University Seoul Korea

**Keywords:** Akt, extracellular signal‐regulated kinase 1/2, fibroblast growth factor receptor, imidazopurine, lung fibrosis, transforming growth factor‐β

## Abstract

Pulmonary fibrosis (PF) is chronic and irreversible damage to the lung characterized by fibroblast activation and matrix deposition. Although recently approved novel anti‐fibrotic agents can improve the lung function and survival of patients with PF, the overall outcomes remain poor. In this study, a novel imidazopurine compound, 3‐(2‐chloro‐6‐fluorobenzyl)‐1,6,7‐trimethyl‐1H‐imidazo[2,1‐*f*]purine‐2,4(3*H*,8*H*)‐dione (IM‐1918), markedly inhibited transforming growth factor (TGF)‐β‐stimulated reporter activity and reduced the expression of representative fibrotic markers, such as connective tissue growth factor, fibronectin, collagen and α‐smooth muscle actin, on human lung fibroblasts. However, IM‐1918 neither decreased Smad‐2 and Smad‐3 nor affected p38MAPK and JNK. Instead, IM‐1918 reduced Akt and extracellular signal‐regulated kinase 1/2 phosphorylation increased by TGF‐β. Additionally, IM‐1918 inhibited the phosphorylation of fibroblast growth factor receptors 1 and 3. In a bleomycin‐induced murine lung fibrosis model, IM‐1918 profoundly reduced fibrotic areas and decreased collagen and α‐smooth muscle actin accumulation. These results suggest that IM‐1918 can be applied to treat lung fibrosis.

## 1 INTRODUCTION

Pulmonary fibrosis (PF) is an incurable and devastating disease represented by destruction and progressive scarring of the lungs with excess connective tissue and extracellular matrix (ECM) deposition. This disease causes respiratory disorder through irreversible loss of the ability to conduct oxygen exchange and eventually leads to death.^1^, ^2^ Idiopathic pulmonary fibrosis (IPF), which is one of the more than 200 types of PF with unknown causes, was initially recognized as an inflammatory disease but was recently considered as being associated with abnormal epithelial cells that activate myofibroblasts and induce ECM remodelling through the secretion of several factors.^1^, ^3^ These secretory molecules include transforming growth factor‐beta (TGF‐β), connective tissue growth factor (CTGF), tumour necrosis factor, platelet‐derived growth factor, osteopontin, angiotensinogen, several matrix metalloproteinases and monocyte chemotactic protein 1, among others.^1^ Therefore, first‐line treatments for IPF have shifted from immunosuppressive drugs such as prednisone (a corticosteroid) or azathioprine (an immunosuppressive) to pirfenidone (a pyridinone derivative) or nintedanib (a multi‐target tyrosine kinase inhibitor) which target these fibrotic growth factors and their receptors, such as TGF‐β1,^4^, ^5^, ^6^ fibroblast growth factor receptors (FGFR) 1‐3, platelet‐derived growth factor receptor α and β and vascular endothelial growth factor receptors 1‐3,^7^ and have received approval from the US Food and Drug Administration in 2014 for treating patients with IPF.^8^ While these therapies provide a significant milestone in IPF treatment, they show some limitations and slow disease progression but do not stop or cure the disease.^9^ Therefore, targeted therapies for IPF based on the cellular and molecular mechanisms of its pathogenesis are needed.

Other TGF‐β‐signalling target inhibitors such as fresolimumab (GC‐1008) and thalidomide are currently being evaluated in clinical trials.^3^, ^10^ TGF‐β is a potent pro‐fibrotic cytokine for which three isoforms have been identified in mammals: TGF‐β1, TGF‐β2 and TGF‐β3. Among these, TGF‐β1 is most closely associated with IPF pathogenesis.^11^ During IPF development, secreted TGF‐β recruits macrophages and fibroblasts to the wound site and activates fibroblasts. It also provokes the differentiation of fibroblasts to activated myofibroblasts, affecting the production and accumulation of excessive ECM.^11^ In contrast, TGF‐β1 is also a well‐known anti‐inflammatory and immunosuppressive factor, and thus approaches aimed at inhibiting TGF‐β1 for IPF treatment have been attempted with caution. Recent studies demonstrated that the roles of inflammatory cells are less critical than the therapeutic effect of TGF‐β1 signalling inhibition,^11^, ^12^ promoting continuous efforts to develop new TGF‐β signalling inhibitors for treating patients with IPF.

In our previous study, we screened chemical libraries using a TGF‐β1‐responsive luciferase‐reported assay system and isolated the imidazopurine compound IM‐412 among several candidates. IM‐412 suppressed TGF‐β‐induced fibroblast differentiation via inhibition of both Smad and non‐Smad signalling pathways in human normal lung fibroblast.^13^ In addition, IM‐412 inhibited invasion and migration of MDA‐MB‐231 breast cancer cells by suppression of epithelial‐to‐mesenchymal transition (EMT) process.^14^ The pharmacological activity of the imidazole moiety has been demonstrated in many medications, including anti‐infective, anticancer, antiviral, antitubercular, anticonvulsant and antidepressant activity^15^ and imidazo[2,1‐*f*]purine‐2,4‐dione derivatives exhibited adenosine receptor antagonist^16^ and potent activator of serotonin transporter.^17^, ^18^ However, molecular target of imidazopurine compounds and their roles in fibrotic process were not clearly elucidated. Here, we investigated whether another analogue of IM‐412, 3‐(2‐chloro‐6‐fluorobenzyl)‐1,6,7‐trimethyl‐1H‐imidazo[2,1‐*f*]purine‐2,4(3*H*,8*H*)‐dione (IM‐1918), inhibits the TGF‐β‐mediated fibrotic process and also evaluated the underlying mechanisms.

## 2 MATERIALS AND METHODS

### 2.1 Agents and Antibodies

Primary antibodies against the following molecules were purchased from commercial suppliers: p‐Smad2 (Ser245/250/255), Smad2, p‐Smad3, Smad3, p‐p38MAPK, p‐Akt, Akt, p‐Erk1/2 and p‐Src (Cell Signaling Technology); CTGF, fibronectin, pro‐collagen, p38MAPK, Erk1/2 and c‐Src (Santa Cruz Biotechnology); α‐SMA (Sigma); p‐FGFR3, FGFR3, p‐FGFR1 and FGFR1 (Abcam); β‐actin (Sigma‐Aldrich); and GAPDH (AbFrontier). Horseradish peroxidase‐conjugated secondary antibodies were obtained from Thermo Fisher Scientific. Recombinant human TGF‐β1 and FGF‐basic were purchased from R&D Systems and Peprotech. IM‐1918 (C_17_H_15_ClFN_5_O_2_, MW: 376; ID 9082937) was purchased from ChemBridge Corporation. The FGFR inhibitor AZD4547 was obtained from Selleckchem.

### 2.2 Cell culture and siRNA transfection

Normal human lung fibroblast CCD18‐Lu, IMR90 and WI38 cells and human embryonic kidney HEK293 cells were purchased from American Type Culture Collection. The cells were maintained at 37°C in minimum essential media or Dulbecco's modified Eagle medium supplemented with 10% foetal bovine serum (Gibco BRL), 100 U/mL penicillin and 100 μg/mL streptomycin in a 5% CO_2_ incubator. CCD18‐Lu cells were transfected with 10 nmole of scramble RNA or siFGFR3 using Lipofectamine RNAiMAX transfection reagent (Invitrogen) according to the manufacturer's instructions. After stabilization for 24 hours, the cells were assayed. The following RNA pairs were used: FGFR3, 5′‐UGA AAG ACG AUG CCA CUG ACA UU‐3′ (forward) and 5′‐UGU CAG UGG CAU CGU CUU UCA UU‐3′ (reverse).

### 2.3 Luciferase reporter assay

For the 3TP‐Lux reporter assay, HEK293 cells were transfected with the 3TP‐Lux plasmid using Lipofectamine 2000 (Invitrogen). The cells were treated with IM‐1918 2 hours prior to adding human recombinant TGF‐β1 (1 ng/mL) and incubated for 24 hours. Luciferase activity was assessed using a microplate reader (Wallac Victor, Perkin‐Elmer). Relative luciferase activity was normalized against cell viability.

### 2.4 Cell viability assessment

Cell viability was determined by MTT (3‐[4,5‐dimethylthiazol‐2‐yl]‐2,5‐diphenyltetrazolium bromide) assays (Sigma‐Aldrich) or lactate dehydrogenase (LDH) assay. Cells (8 × 10^3^) were seeded into 24‐well plates (SPL Life Sciences) and stabilized for 24 hours. The indicated dose of IM‐1918 was added to each well, and the plate was incubated for 24 hours. MTT (0.5 mg/mL) reagent was added for further incubation for 4 hours, and absorbance was measured at 540‐nm using a microplate reader (Multiskan EX, Thermo LabSystems). The release of LDH in culture medium was measured with a commercial assay kit (Dojindo Molecular Technologies, Inc), and absorbance was detected at 490‐nm. The experiment was repeated three times. The GI_50_ value was calculated by GraphPad Prism 5 software (GraphPad, Inc).

### 2.5 Western blot analysis

To analyse total proteins, cells or lung tissues were lysed with RIPA buffer (50 mM Tris–Cl, pH 7.4, 1% NP‐40, 150 mM NaCl, 1 mM EDTA) supplemented with protease inhibitors (1 mM phenylmethylsulfonyl fluoride, 1 μg/mL aprotinin and 1 μg/mL leupeptin) and phosphatase inhibitors (1 mM Na_3_VO_4_ and 1 mM NaF). Protein samples were separated by SDS‐polyacrylamide gel electrophoresis and transferred to nitrocellulose membranes (Bio‐Rad). After blocking non‐specific antibody sites, the membranes were probed overnight at 4°C with primary antibodies. The membranes were incubated with peroxidase‐conjugated secondary antibodies, and immunoreactive bands were visualized by enhanced chemiluminescence reagents (GE Healthcare). The experiments were repeated at least three times.

### 2.6 Bleomycin‐induced pulmonary fibrosis mouse model

Six‐week‐old male C57BL/6 mice were purchased from Dae‐Han Laboratory Animal Research Co. (Daejeon, Korea) and housed at 50 ± 10% humidity and 22 ± 2°C with free access to sterile food and water. After acclimatization for 1 week, the mice were randomly distributed into experimental groups (n = 5). The mice were anesthetized with ketamine (100 mg/kg) and xylazine (10 mg/kg), and then intratracheally administered an instillation of 100 mg/kg bleomycin (BLM; Nippon Kayaku Co.) through a 27‐gauge needle.^19^ IM‐1918 (2 mg/kg) or vehicle (0.1% dimethyl sulfoxide) was intraperitoneally injected every other day, starting on day 1 after BLM treatment. On day 14 after BLM administration, the mice were sacrificed, and the harvested lungs were subjected to immunohistochemical analysis and western blotting. All animal experiments were approved by the Institutional Animal Care and Use Committee of the Korea Institute of Radiological and Medical Sciences.

### 2.7 Histological analysis and immunohistochemical staining

The lung tissues were fixed in formalin for 24 hours and embedded in paraffin (Hayashi Pure Chemical Industries). Left lung tissue sections (3 µm) were cut and stained with haematoxylin and eosin or Masson's trichrome for histopathological examination and evaluation of collagen accumulation. The dewaxed sections were exposed to 3% H_2_O_2_ for 10 minutes to block endogenous peroxidase activity, followed by incubation with primary antibody against α‐SMA for 60 minutes at room temperature using a Cap‐Plus kit (Zymed) according to the manufacturer's protocol. The sections were then incubated with the biotinylated secondary antibody for 40 minutes, and streptavidin conjugate successively for 30 minutes at room temperature. After three washes (5 minutes each) with PBS‐Tween 20, the slides were exposed to diaminobenzidine solution and counterstained with Mayer's haematoxylin. The sections were mounted in Permount (Thermo Fisher Scientific), and images were obtained using a microscope.

### 2.8 Data analysis

Data are represented as means ± SD. Significant differences between groups were determined by analysis of variance and Tukey's post hoc comparisons using GraphPad software version 5. Statistical significance was defined as *P*‐values < .05.

## 3 RESULTS

### 3.1 Imidazopurine derivative IM‐1918 reduces TGF‐β‐induced fibrotic process

**Figure 1:**
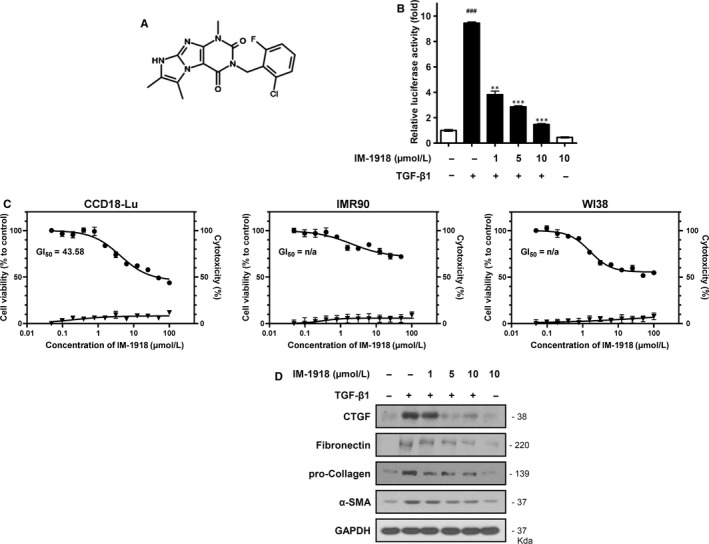
IM‐1918 decreases the TGF‐β‐responsive reporter activity. A, Schematic structure of an imidazopurine derivative, 3‐(2‐chloro‐6‐fluorobenzyl)‐1,6,7‐trimethyl‐1H‐imidazo[2,1‐*f*]purine‐2,4(3*H*,8*H*)‐dione (IM‐1918). B, HEK293 cells were transiently transfected with 3TP‐Lux reporter gene and then seeded into 96‐well plates. After 24 h, IM‐1918 was added 2 h prior to adding human recombinant TGF‐β1 (1 ng/mL) and incubated for 24 h. Data shown are the means ± SD of three independent experiments. ^†††^ *P* < .001 vs control, **P < .01 and ***P < .001 vs TGF‐β1‐alone treatment. C, MTT and Lactate dehydrogenase (LDH) assays were performed to determine cell viability (circles, left *y*‐axis) and cytotoxicity (triangles, right *y*‐axis), respectively. Cells were seeded at 8 × 10^3^ cells/well into 24‐well plates and treated with the indicated concentrations of IM‐1918 for 24 h. Data shown are the means ± SD of three independent experiments. D, CCD18‐Lu cells were treated with the indicated dose of IM‐1918. Two hours later, TGF‐β1 (1 ng/mL) was added, and the cells were incubated for 24 h. Total protein was isolated and analysed by Western blotting for the indicated proteins

To develop a novel inhibitor of TGF‐β‐mediated fibrosis, we previously performed cell‐based screening of chemical libraries using a reporter assay of a 3TP‐Lux construct stably transfected into HEK293 cells.^13^ Among the active candidate compounds, the imidazopurine derivative IM‐1918 was identified to be active and inhibit the TGF‐β‐induced response (Figure 1A,B). IM‐1918 dose‐dependently inhibited TGF‐β‐mediated luciferase activity (IC_50_ = 4.28 μM). To determine whether IM‐1918 inhibits TGF‐β1‐induced cellular responses in lung fibroblast cells, the effect of IM‐1918 on cell viability was measured. As shown in Figure 1C, the viability of CCD18‐Lu cells was weakly decreased by IM‐1918, and the concentration at which 50% inhibition of cell growth occurred (GI_50_) was 43.58 μM. The GI_50_ values of IM‐1918 for IMR‐90 and WI‐38 cells could not be determined. TGF‐β treatment did not affect cell viability of CCD18‐Lu, and the cell viability‐induced by IM‐1918 was not altered in the presence or absence of TGF‐β (Figure S1A,B). In addition, IM‐1918 was not shown to be cytotoxic in lung fibroblasts (Figure 1C). Next, the expression of fibrosis‐associated TGF‐β1 target molecules was determined. The levels of CTGF, fibronectin, pro‐collagen I and α‐SMA expression were remarkably increased by TGF‐β1 treatment, whereas IM‐1918 significantly decreased these proteins in a dose dependent manner (Figure 1D). These data indicate that IM‐1918 effectively reduced the TGF‐β1‐induced fibrotic process without causing cytotoxicity.

### 3.2 IM‐1918 suppresses non‐canonical TGF‐β signalling pathways

**Figure 2:**
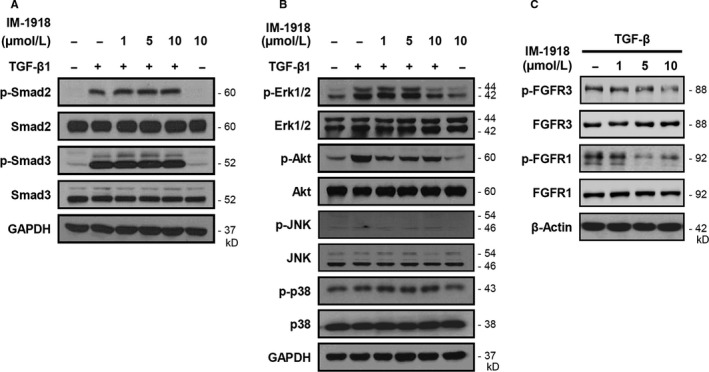
IM‐1918 inhibits TGF‐β‐induced fibrotic response via Erk1/2 and AKT down‐regulation. CCD18‐Lu cells were treated with the indicated dose of IM‐1918. Two hours later, TGF‐β1 (1 ng/mL) was added, and the cells were incubated for 24 h. Total protein was isolated and analysed by Western blotting for the indicated proteins. A, Smad signalling. B, Non‐Smad signalling. C, FGFR

To investigate the target molecule of IM‐1918 in TGF‐β1‐mediated signalling pathways, we determined the phosphorylation of Smad‐2 and Smad‐3, a canonical TGF‐β1 signalling pathway. Unlike compound IM‐412, which blocked the Smad pathway as we previously described,^13^ IM‐1918 did not inhibit phosphorylation of either Smad2 or Smad3 (Figure 2A). We next investigated whether IM‐1918 could inhibit the Smad‐independent signalling pathway, including Akt, extracellular signal‐regulated kinase (Erk) 1/2, c‐Jun N‐terminal kinase (JNK) and p38 mitogen‐activated protein kinase (p38MAPK). IM‐1918 inhibited the expression of phosphorylated Erk1/2 and Akt but did not alter the phosphorylation of JNK or p38MAPK (Figure 2B).

To clarify the inhibitory activity of IM‐1918 towards target proteins, an in vitro kinase assay was performed (Table 1). Interestingly, most kinases tested showed no significant inhibition by IM‐1918, and only seven kinases were slightly inhibited by approximately 10%. Additionally, IM‐1918 did not directly inhibit Erk1/2, Akt or TGF‐β receptor (TGFBRI, TGFBRII). IM‐1918 showed the highest inhibitory activity towards FGFR3 with 36.09% inhibition, followed by FGFR1 with 18.36% inhibition. Because FGFR3 is structurally similar to FGFR1, particularly at the ATP binding site, the levels of FGFR1/3 and phosphorylated FGFR1/3 were determined. The levels of FGFR3 expression were not altered by IM‐1918, while the phosphorylation level of FGFR3 was decreased by IM‐1918 treatment. IM‐1918 also effectively inhibited the phosphorylation of FGFR1 without altering FGFR1 protein expression (Figure 2C). These results demonstrate that IM‐1918 reserved TGF‐β‐induced Smad signalling cascades, but decreased the expression of fibrosis‐related genes by inhibiting non‐classical Smad signalling pathways through partial suppression of the receptor tyrosine kinase activity of FGFR1/3.

**Table 1:** Activity of IM1918 in an in vitro kinase assay

Kinase	Activity (average, %)	Kinase	Activity (average, %)
FGFR3	63.91 ± 0.185	ERK2/MAPK1	99.5 ± 0.695
FGFR1	81.64 ± 2.185	ALK	99.71 ± 0.556
p70S6K/RPS6KB1	85.35 ± 0.056	GRK2	100.33 ± 1.444
FYN	85.89 ± 1.698	ROS/ROS1	100.54 ± 2.611
P38a/MAPK14	87.79 ± 7.829	JAK3	100.84 ± 0.61
YES/YES1	88.15 ± 1.999	JNK3/MAPK10	100.89 ± 2.331
ZAP70	89.47 ± 2.099	AKT2	101.83 ± 3.632
AKT1	90.26 ± 1.076	FAK/PTK2	101.86 ± 0.26
JAK1	91.38 ± 0.141	TAK1/MAP3K7	101.95 ± 8.525
IGF1R	92.08 ± 1.335	ASK1/MAP3K5	102.25 ± 0.841
IKKb/IKBKB	92.18 ± 2.026	JNK1/MAPK8	102.42 ± 2.462
PKA	93.6 ± 1.914	ERK1/MAPK3	102.6 ± 3.873
PAK2	95.07 ± 1.978	SRC/c‐Src	102.66 ± 2.875
SYK	95.6 ± 3.765	PYK2/PTK2B	102.69 ± 0.918
ROCK1	95.77 ± 1.82	TYK2	102.83 ± 12.29
JNK2/MAPK9	96.35 ± 2.736	PDK1/PDPK1	105.71 ± 2.438
FGFR2	96.77 ± 1.601	TYK1/LTK	106.77 ± 3.737
TYRO3/SKY	96.94 ± 0.975	PAK1	107.06 ± 5.566
JAK2	96.97 ± 8.383	RAF1	107.28 ± 8.001
BRAF	97.74 ± 1.883	IRAK4	109.78 ± 1.391
P38b/MAPK11	98.61 ± 2.143	ARAF	119.05 ± 1.864
TGFBR1/ALK5	98.88 ± 0.483	TGFBR2	134.3 ± 2.88
GSK3b	99.19 ± 5.12		

### 3.3 IM‐1918 inhibits basic fibroblast growth factor (bFGF)‐mediated fibrotic process

**Figure 3:**
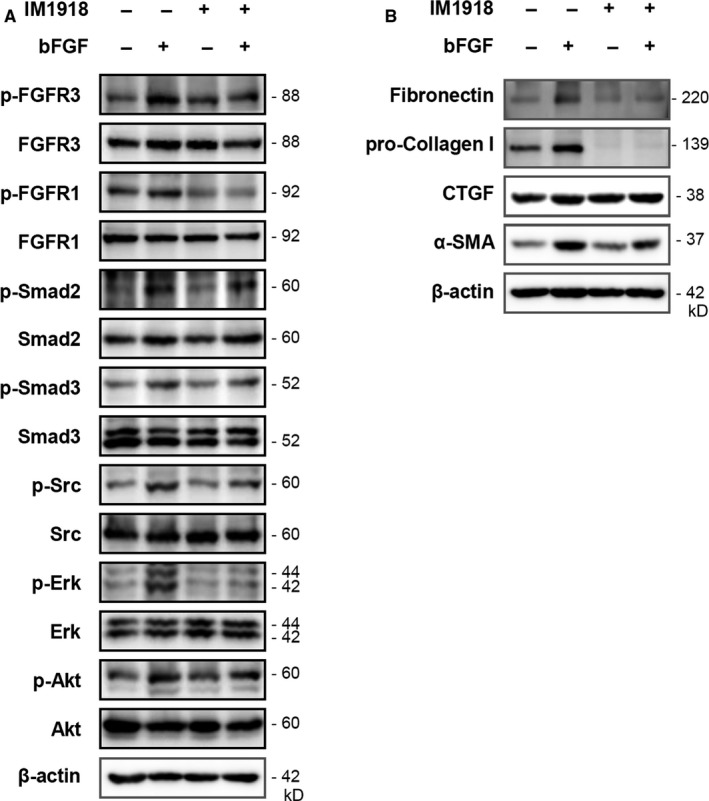
IM‐1918 suppresses the bFGF‐mediated fibrotic process via FGFR inhibition. CCD18‐Lu cells were treated with 10 μM IM‐1918 for 2 h, followed by treatment with bFGF (100 ng/mL) for 5 h. Total protein was isolated and analysed by Western blotting for the indicated proteins. A, Fibrosis‐related signalling molecules. B, Representative fibrotic markers

Because FGF signalling has been implicated in the pathogenesis of PF and co‐operatively cross‐talks with TGF‐β1,^20^ we investigated whether IM‐1918 disrupts the bFGF‐induced fibrotic process. Treatment with bFGF increased the activation of FGFR1/3 and phosphorylation of Akt and Erk1/2 (Figure 3A). In agreement with the above results, IM‐1918 did not directly alter the expression level of fibrosis‐related molecules but attenuated the induction of FGFR1/3, Erk1/2 and Akt following bFGF treatment. Interestingly, the phosphorylation of Smad‐2 and Smad‐3 was increased by bFGF, while these proteins were not suppressed by IM‐1918 treatment. Additionally, we evaluated the effect of IM‐1918 on Src, which is the best‐characterized non‐receptor tyrosine kinase. Src can interact with receptor tyrosine kinases (RTKs) and activate downstream signalling molecules such as Erk1/2 and Akt.^21^, ^22^, ^23^ Moreover, targeting of Src is considered effective for attenuating fibrosis.^24^ As expected, phosphorylation of Src was markedly increased by bFGF, whereas IM‐1918 suppressed this induction.

The bFGF treatment of CCD18‐Lu cells induced about 20% more proliferation than control cells whereas IM‐1918 inhibited bFGF‐induced cell proliferation to the control levels (Figure S1C,D). Treatment with bFGF also enhanced the expression level of fibrotic proteins, including fibronectin, pro‐collagens I, CTGF and α‐SMA (Figure 3B). These up‐regulated proteins were significantly decreased by treatment with IM‐1918, indicating that IM‐1918 attenuated the bFGF‐mediated fibrotic process by suppressing FGFR1/3.

### 3.4 Inhibition of FGFR3 disrupts TGF‐β1‐mediated fibrotic process

**Figure 4:**
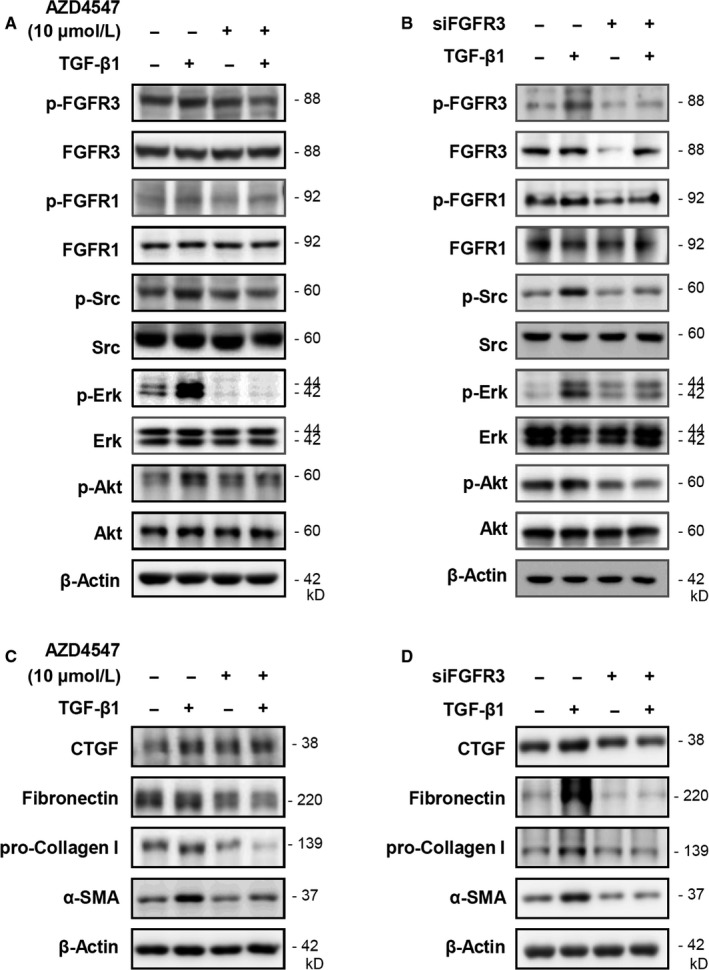
Knockdown of FGFR3 inhibits the expression of TGF‐β1‐induced fibrosis‐associated molecules. CCD18‐Lu cells were treated with 10 μM of AZD4547 for 2 h, following treatment with TGF‐β1 (1 ng/mL) for 5 h. Total protein was isolated and analysed by Western blotting for the indicated proteins. A, Signalling molecules. C, Fibrotic markers. CCD18‐Lu cells were transfected with scramble RNA or siFGFR3, and then incubated for 24 h. TGF‐β1 (1 ng/mL) was added to the cells, and the cells were incubated for an additional 5 h. Total protein was isolated and analysed by Western blotting for the indicated proteins. B, Signalling molecules. D, Fibrotic markers

To verify that inhibition of FGFR3 blocks TGF‐β1‐induced fibrotic activity, pharmacologic inhibition of FGFR3 using AZD4547 treatment or knockdown of FGFR3 by short interfering RNA genetic inactivation was performed. AZD4547, a well‐known pan‐FGFR inhibitor, slightly suppressed the phosphorylation of FGFR3 at the doses tested in this study. Moreover, AZD4547 did not alter fibrotic protein expression, except for decreasing phosphorylated Erk1/2. Similar to the results shown in Figure 2, TGF‐β1 increased downstream signalling molecules, whereas this enhanced expression of fibrotic markers and related signalling proteins were markedly decreased by AZD4547 (Figure 4A,C).

Treatment with siFGFR3 inhibited the levels of FGFR and phosphorylated FGFR3 expression, while FGFR1 proteins were not changed. Additionally, only phosphorylated Akt was decreased by siFGFR3. Nevertheless, siFGFR3 effectively suppressed TGF‐β1‐increased fibrotic activity, which was similar to the results of AZD4547 treatment (Figure 4B,D). These results indicate that pharmacological and genetic inhibition of FGFR3 can suppress the TGF‐β1‐mediated fibrotic process. These results indicate that inhibition of FGFR3 under normal conditions (without TGF‐β1 or bFGF stimuli) can be easily and rapidly compensated by one of the other subtypes of FGFR or by redundant downstream signalling molecules.

### 3.5 IM‐1918 attenuates BLM‐induced murine pulmonary fibrosis

**Figure 5:**
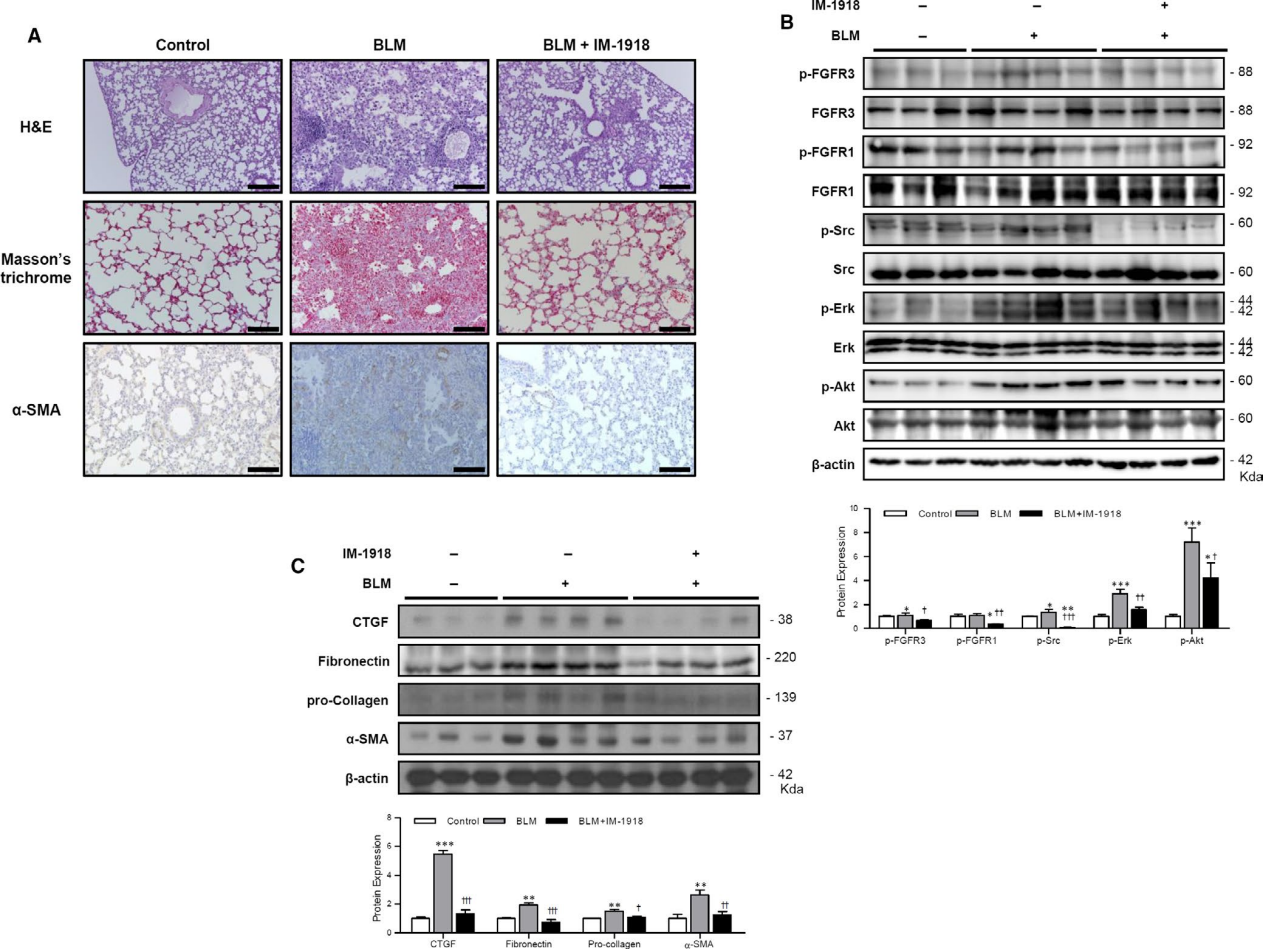
IM‐1918 attenuates the bleomycin‐induced fibrotic process. Lungs from C57BL/6 mice were harvested on day 14 after intratracheal administration of bleomycin (BLM, 100 mg/kg). IM‐1918 (2 mg/kg) or vehicle (0.1% DMSO, control) was intraperitoneally injected e.o.d starting 24 h after BLM treatment. A, Lung sections stained with haematoxylin and eosin for morphological evaluation (top), Masson's Trichrome (middle) to detect collagen deposition and immunohistochemical detection of α‐SMA (bottom). Representative images are shown. Magnification ×100. Scale bar = 100 μm. B, C, Expression of fibrotic markers or related signalling molecules in lung tissues of experimental mice was analysed by Western blotting for the indicated proteins. Band intensities corresponding to the indicated proteins were quantified by densitometry using ImageJ software, normalized to β‐actin or the total form of each protein, and expressed as the fold‐change compared to each control. Data were considered significant at *P < .05, **P < .01, ***P < .001 vs control; ^†^ *P* < .05, ^††^ *P* < .01, ^†††^ *P* < .001 vs BLM

**Figure 6:**
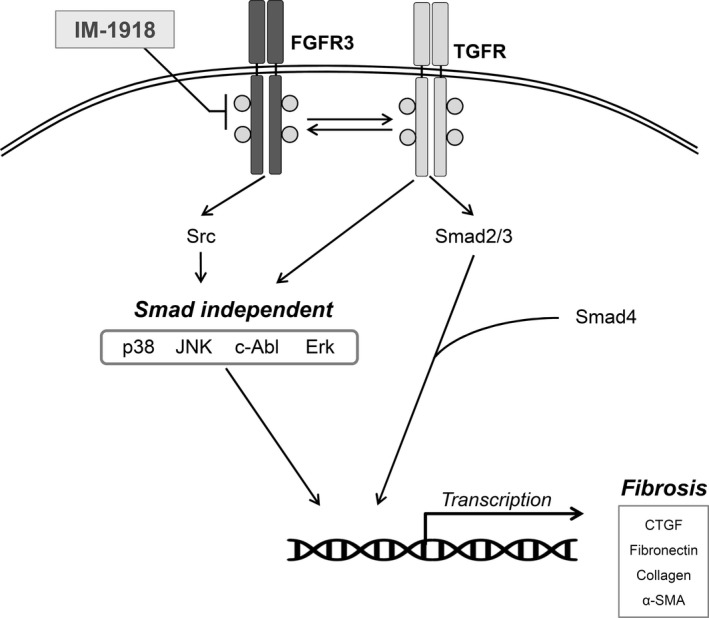
Schematic model for anti‐fibrotic activity of IM‐1918. TGF‐β is a well‐known mediator that promotes the fibrotic process via Smad‐dependent and Smad‐independent pathways. Treatment with bFGF also induced activation of fibrotic molecules, in co‐operation with TGF‐β1. IM‐1918 prevents phosphorylation and activation of FGFR3, leading to attenuation of the fibrotic process in vitro and in vivo

To investigate the efficacy of IM‐1918 on fibrosis in animals, a murine BLM‐induced lung fibrosis model was used. Immunohistochemical analysis revealed that BLM‐induced accumulation of collagen, and α‐SMA was markedly inhibited by IM‐1918 (Figure 5A). Furthermore, several signalling molecules and fibrotic markers investigated by in vitro analysis were significantly increased in the lung tissues of BLM‐treated mice (Figure 5B,C). In accordance with the above results, increased expression of proteins involved in the BLM‐induced fibrotic process was markedly attenuated by administration of IM‐1918. These results demonstrate that IM‐1918 can inhibit PF both in vivo and in vitro. Based on these findings, the molecular mechanisms of the anti‐fibrotic effect of IM‐1918 are schematically illustrated in Figure 6.

## 4 DISCUSSION

TGF‐β is a major causative factor of symptoms of fibrotic diseases and multifunctional cytokine that plays various roles in the body.^12^ In this study, the novel compound IM‐1918 effectively suppressed the expression of fibrosis‐related molecules up‐regulated by TGF‐β1 in a Smad‐independent manner. Although we identified IM‐1918 in a TGF‐β1‐responsive reporter assay, the putative primary target of IM‐1918 is FGFR3 based on data from an in vitro kinase assay. Most kinases, including TGFBRI, TGFBRII, EGFR, AKT, p38MAPK, ERK1/2, JNK, RAF and JAK, among others, were not affected by IM‐1918.

Several studies have demonstrated that administration of TGF‐β1 changes the sensitivities of FGFRs and activates the production of FGF‐2 in primary human lung fibroblasts.^25^, ^26^, ^27^ FGFs are associated with the pathogenesis of PF, and FGF2‐neutralizing antibodies successfully inhibit the TGF‐β1‐mediated fibrotic process.^20^ It has been also reported that FGF‐2 and FGFR1IIIc are involved in EMT and advanced cancer progression, which may be regulated by TGF‐β1 autonomously secreted from cancer cells.^25^, ^28^ Moreover, non‐selective inhibition of RTKs and non‐specific inhibition of FGFRs decrease BLM‐induced PF in rodents.^7^, ^29^ Altered expression of FGFR1 and FGF1 proteins was observed in the lungs of patients with IPF,^30^ and a specific FGFR1 inhibitor (NP603) attenuated carbon tetrachloride‐induced hepatic fibrosis in rats.^31^ In addition to FGF, epidermal growth factor, which acts through RTKs, can synergize with TGF‐β signalling to increase collagen accumulation and interstitial fibrosis,^32^, ^33^ suggesting that a co‐operative network exists between RTKs and TGF‐β1. In agreement with these observations, administration of TGF‐β1 to CCD18‐Lu fibroblasts increased the phosphorylation of FGFR3 and FGFR1, accompanied by induction of ECM proteins in this study. Although all FGFR1‐4 were expressed in freshly isolated lung mesenchyme, only FGFR1 was expressed when isolated fibroblasts are cultured.^20^ In addition, low expression levels of FGFR3 and FGFR4 in cultured lung fibroblasts was also reported.^34^ Therefore, we cautiously propose that although the inhibitory activity of IM‐1918 on FGFR3 was about two‐fold higher than on FGFR1, FGFR1 expression in lung fibroblasts was much higher than that of FGFR3, allowing clear exhibition of FGFR1 inhibition. Nevertheless, the redundancy of FGFs/FGFRs allows reciprocal compensation of the deficiency of each and crosstalk with TGF‐β1 signalling utilizes the same and similar downstream effectors, such as Src and Erk activation to maintain their biological functions. In this regard, it is worth that IM‐1918 effectively inhibited the TGF‐β1 or bFGF‐induced fibrotic process by suppression of Src/Erk signalling as well as ECM accumulation. However, the exact types of FGFs/FGFRs and isoforms of FGFRs, which are critical targets for FGFs/FGFRs, interactions of related receptors, and cellular mechanisms involved in fibrosis remain unclear. This is because these factors are highly dependent on the cells or experiments used and the degree of differentiation, and further studies are continually required to identify individual roles of each FGFRs and specific intermediate signals in pulmonary fibrosis. In contrast, although BLM‐induced PF does not perfectly resemble the pathology and chronicity of human PF, BLM remains a well‐established and useful model for studying PF.^35^, ^36^ In vivo IM‐1918 administration significantly decreased CTGF, fibronectin, α‐SMA and collagen accumulation in a BLM‐induced lung fibrosis animal model, suggesting that IM‐1918 is a highly efficacious anti‐fibrotic agent with potential for further clinical application.

Recently, an important study showed that the relative amounts of FGF‐2 and TGF‐β determine the invasive potential by FGFR substrate (FRS2) regulation in medulloblastoma.^37^ Upon ligand (FGF) binding to FGFR, dimerization causes the receptors to rapidly auto‐transphosphorylate several tyrosine residues, leading to activation of downstream molecules, such as FRS2 and phospholipase C‐gamma.^38^, ^39^ Activated FRS2 triggers the Ras/MAPK kinase signalling pathway.^40^, ^41^ FRS2 also has been shown to recruit Src, which regulates numerous signalling cascades involved in cell viability, proliferation, differentiation, migration and metabolism.^42^, ^43^, ^44^ We revealed that IM‐1918 decreased PI3K/Akt and Erk1/2 activity in non‐classical TGF‐β1 pathways. Additionally, clear inhibition of TGF‐β1‐induced fibroblast differentiation was demonstrated when the cells were exposed to IM‐1918, the pan‐FGFR inhibitor AZD4547 and siFRFG3. These effects of IM‐1918 involve concurrent disruption of TGF‐β1‐ and FGFR3‐mediated signalling pathways converged on the Src‐Akt/Erk axis.

In summary, we demonstrated that the interplay between TGF‐β1 and FGFR3 may promote fibrotic disease in human lung fibroblasts. Additionally, the new small molecule IM‐1918 significantly inhibits the TGF‐β1‐mediated fibrotic process by suppressing the FGFR3‐Src‐Akt/Erk signalling pathway. Although abundant feedback and parallel signalling pathways can limit the efficacy of this compound or the value of the targets, such as FGFs, FGFRs and Src, additional studies are needed to develop a successful strategy for treating patients with PF.

## CONFLICT OF INTEREST

The authors declare that they have no competing interests.

## AUTHOR CONTRIBUTIONS

M‐H Kim, S‐Y Jung, K‐H Song and J‐I Park performed the research. M‐H Kim performed the animal experiment. M‐H Kim, S‐Y Jung, H‐J Woo and J‐Y Song designed the research study. M‐H Kim, S‐Y Jung, K‐H Song and J‐Y Song analysed the data. J. Ahn, E‐H Kim, JK Park, S‐G Hwang and H‐J Woo participated in discussion. M‐H Kim, S‐Y Jung and J‐Y Song wrote the paper.

## Data Availability

The data that support the findings of this study are available from the corresponding author upon reasonable request.
