# Supplementary figures and images for: A new FGFR inhibitor disrupts the TGF‐β1‐induced fibrotic process
Source: J Cell Mol Med. 2019 Nov 6;24(1):830–40. doi: 10.1111/jcmm.14793 (PMC6933341; doi:10.1111/jcmm.14793)

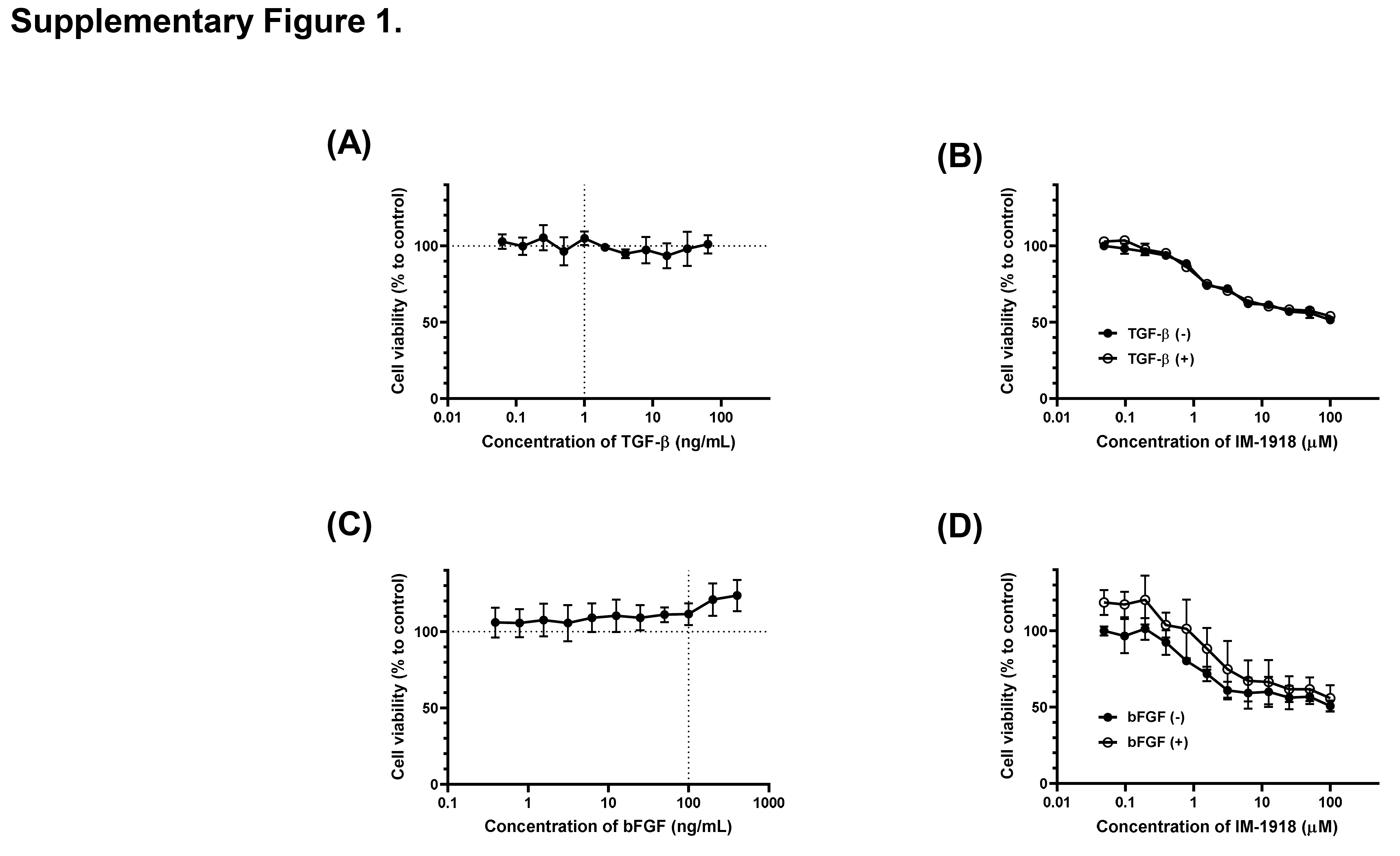

Supplement: Supplementary file 1 [file JCMM-24-830-s001.tif]
