# Supplementary material for: A new FGFR inhibitor disrupts the TGF‐β1‐induced fibrotic process
Source: J Cell Mol Med. 2019 Nov 6;24(1):830–40. doi: 10.1111/jcmm.14793 (PMC6933341; doi:10.1111/jcmm.14793)
Supplement: Supplementary file 2 [file JCMM-24-830-s002.docx]

**Supplementary Information**

**Supplementary Figure 1.** Effect of IM-1918 on TGF-β1 or bFGF-induced proliferation of human lung fibroblasts. CCD18-Lu cells were treated with the indicated dose of TGF-β1 (A) or bFGF (C) for 24 h, and MTT assay performed to determine proliferative response. (B) CCD18-Lu cells were treated with the indicated dose of IM-1918 for 2 h prior to adding 1 ng/mL TGF-β1 (B) or 100 ng/mL bFGF (D). After 24 h incubation, cell viability was determined by MTT assay. Data shown are means ± SD.
